# Minocycline Inhibits Microglial Activation and Improves Visual Function in a Chronic Model of Age-Related Retinal Degeneration

**DOI:** 10.3390/biomedicines10123222

**Published:** 2022-12-12

**Authors:** Xuan Du, Eimear M. Byrne, Mei Chen, Heping Xu

**Affiliations:** The Wellcome-Wolfson Institute for Experimental Medicine, School of Medicine, Dentistry & Biomedical Sciences, Queen’s University, Belfast BT9 7BL, UK

**Keywords:** ageing, inflammation, neurodegeneration, immunomodulation, age-related macular degeneration, neuroprotection

## Abstract

Age-related macular degeneration (AMD) is a chronic disease, which progresses slowly from early to late stages over many years. Inflammation critically contributes to the pathogenesis of AMD. Here, we investigated the therapeutic potential of minocycline in a chronic model of AMD (i.e., the *LysMCre-Socs3^fl/fl^Cx3cr1^gfp/gfp^* double knockout [DKO] mice). Five-month-old DKO and wild type (WT) (*Socs3^fl/fl^*) mice were gavage fed with minocycline (25 mg/kg daily) or vehicle (distilled water) for 3 months. At the end of the treatment, visual function and retinal changes were examined clinically (using electroretinography, fundus photograph and optic coherence tomography) and immunohistologically. Three months of minocycline treatment did not affect the body weight, behaviour and general health of WT and DKO mice. Minocycline treatment enhanced the a-/b-wave aptitudes and increased retinal thickness in both WT and DKO. DKO mouse retina expressed higher levels of *Il1b*, *CD68* and *CD86* and had mild microglial activation, and decreased numbers of arrestin^+^ photoreceptors, PKCα^+^ and secretagogin^+^ bipolar cells compared to WT mouse retina. Minocycline treatment reduced microglial activation and rescued retinal neuronal loss in DKO mice. Our results suggest that long-term minocycline treatment is safe and effective in controlling microglial activation and preserving visual function in chronic models of AMD.

## 1. Introduction

Retinal degenerative diseases, including age-related macular degeneration (AMD), retinitis pigmentosa (RP), diabetic retinopathy (DR) and glaucoma, are responsible for a large proportion of blindness [1,2,3,4,5,6]. Although the initial causes differ in the various retinal degenerative conditions, inflammation is known to play a critical role in disease progression in all cases [7,8,9,10,11]. Based on whether the blood-retinal barrier (BRB) is damaged and fluid accumulation exists, we recently proposed to classify retinal degeneration into “dry” and “wet” forms. Indeed, immunosuppressive drugs such as dexamethasone and fluocinolone have been successfully used to control symptoms in “wet” forms of retinal degeneration [12,13,14], highlighting the pathogenic role of inflammation in these conditions.

Inflammation is a protective response to tissue damage. A well-regulated immune response can remove debris and promote repair. When inflammation is severe or dysregulated, it becomes detrimental, causing collateral damage. Targeting specific disease-causing pathways would be ideal, although identifying the target remains a big challenge. In “dry” retinal degeneration, the BRB is relatively intact, and inflammation is executed predominately by microglia and the intraocular complement system and at low levels [11]. The disease often progresses slowly, and the deterioration of visual function may last many years [11]. Global suppression of inflammation using steroids is effective in controlling symptoms in “wet” retinal degeneration, however this approach is inappropriate to treat “dry” retinal degeneration due to its severe adverse effects both systemically (e.g., infection, hypertension, cancer, etc.) and locally (e.g., glaucoma, cataract, etc.). Because dry retinal degeneration needs long-term management, modulating inflammation along with neuroprotection would be a clinically applicable approach to slow disease progression.

Minocycline was originally synthesized as an antibiotic. However, around two decades ago, minocycline was discovered to have an inhibitory effect on microglial activation as well as a novel neuroprotective effect [15]. Since then, minocycline has been investigated extensively in a variety of in vitro studies and in animal models of neurodegenerative diseases including stroke, Alzheimer’s disease, Parkinson’s disease, multiple sclerosis, spinal cord injury and traumatic brain injury [16,17]. Minocycline has been shown to be anti-inflammatory and neuroprotective in various retinal degenerative conditions, including branch retinal vein occlusion [18], DR [19], ischemia-reperfusion mediated retinal degeneration [20,21], light-induced retinal degeneration [22], and inherited retinal degeneration model [23]. It has been shown that short-term (up to two weeks) treatment with minocycline reduces microglial activation and rescues retinal neuronal degeneration [19,20,21,22,23]. However, retinal degeneration in clinical settings requires long-term (e.g., months~years) management. It is, therefore, necessary to assess the therapeutic potential of minocycline in chronic models of retinal degeneration.

We previously reported a mouse model of age-related retinal microgliopathy, whereby, two immune checkpoint genes *Cx3cr1* and *Socs3* were deleted in microglial cells (i.e., the *LysMCre-Socs3^fl/fl^Cx3cr1^gfp/gfp^* double knockout (DKO) mice) leading to uncontrolled microglial activation, retinal neuron and RPE degeneration when mice reach 10~12 months of age [24]. The DKO mice, therefore, are an ideal model to test the long-term therapeutic potential of minocycline.

## 2. Materials and Methods

### 2.1. Animals

The *LysMCre-Socs3^fl/fl^Cx3cr1^gfp/gfp^* DKO mouse line was generated by crossing bred the *LysMCre-Socs3^fl/fl^* mice with the *Cx3cr1^gfp/gfp^* mice (all in C57BL/6J background) as described previously by us [24]. The *Socs3^fl/fl^* mice were used as wild-type (WT) controls in all experiments. All mice were maintained in the biological resource unit at Queen’s University Belfast and exposed to a 12 h light/dark cycle with free access to water and food. All procedures were conducted under the regulation of the UK Home Office Animals (Scientific Procedures) Act 1986 and approved by the Animal Welfare & Ethical Review Body of Queen’s University Belfast.

### 2.2. Study Design and Minocycline Administration

Experimental animals were randomized into 4 groups, (A) *Socs3^fl/fl^* + vehicle (*n* = 11, 4 males and 7 females); (B) *Socs3^fl/fl^* + minocycline (*n* = 10, 6 males and 4 females); (C) DKO + vehicle (*n* = 16, 8 males and 8 females); (D) DKO + minocycline (*n* = 12, 5 males and 7 females). When mice reached 5 months of age, animals in groups A and C were given distilled water throughout the experiment and served as controls. Group B and D were administered with minocycline (Glentham Life Sciences, Corsham, UK) at a dose of 25 mg/kg daily by oral gavage for 3 months (volume: 100 μL/25 g body weight). In vivo investigation was carried out to study the function of the retina 24 h after the last minocycline administration. Eyes were then collected for further in vitro investigations.

### 2.3. Electroretinography (ERG)

Scotopic electroretinogram (ERG) responses were evaluated in all groups of experimental animals (*n* = 8). Mice were dark-adapted overnight, and all procedures were conducted under dim-red light (<1 Lux). Briefly, mice were anaesthetized via intraperitoneal injection of ketamine hydrochloride (60 mg/kg; Vetoquinol UK Ltd., Northamptonshire, UK) and xylazine hydrochloride (5 mg/kg; Pharmacia & Veterinary Products, Kiel, Germany). Pupils were dilated with 1% tropicamide and 2.5% phenylephrine (Chauvin, Essex, UK). The mouse was placed on a homeothermic pad at 38 °C (Kobayashi Healthcare, London, UK). ERG responses were recorded using corneal ERG electrodes placed on each eye in response to a single white light flash, delivered by a standard Ganzfeld Stimulator (LKC Technologies, Gaithersburg, MD, USA). Eight light intensities ranging from 0.008 to 25 cd·s/m^2^ were used. The a-wave and b-wave amplitudes at each intensity were averaged and shown. Oscillatory potentials (OPs) were recorded at 25 cd·s/m^2^ (amplitudes of wavelets 2–5 were summed for statistical analysis).

### 2.4. Fundus Photography and Micron IV Examination

Fundus images were obtained after anesthesia and pupil dilation of the mice described previously [24]. Images were captured with a Nikon D90 camera via an endoscope or the Micron IV system (Phoenix Research Labs, Pleasanton, CA, USA). Retinal green fluorescent images in DKO mice were acquired using the Micron IV system. The illumination settings and the gain were consistent in each subject. Images were saved in TIFF format.

### 2.5. Spectral Domain Optical Coherence Tomography (SD-OCT)

Spectralis Heidelberg OCT system (Heidelberg Engineering, Heidelberg, Germany) was used for SD-OCT investigation, according to manufacturers’ instructions. Animals were anaesthetized and pupils were dilated as described above. At least 8 animals per group were analysed. The retinal thickness was measured manually, and the measurement was kept consistent. The thicknesses of the neural retina and the photoreceptor layer were also measured. The thickness data was plotted separately and averaged.

### 2.6. Reverse Transcription and Real-Time aRT-PCR

Total RNA was extracted from retinal tissues by RNeasy Mini kit (Qiagen Ltd., Crawley, UK) according to the manufacturer’s instructions. The quantity and quality of RNA were determined using a NanoDrop ND-1000 spectrophotometer (NanoDrop Technologies, Wilmington, DE, USA). The same amount of total RNA was used for reverse transcription using SuperScrip^TM^ II Reverse Transcriptase kit and random primers (Invitrogen). Real-time RT-PCR was performed using SYBR Green Master in LightCycler^®^ 480 system (Roche Diagnostics GmbH, Mannheim, Germany). The primer sequences are listed in Table 1. Beta-actin (*Actb*) was used as housekeeping control. RNA from 6 retinas per group was analysed.

### 2.7. Retinal and RPE/Choroidal Flat Mount Preparation

The eyes were collected and fixed with 2% paraformaldehyde for 2 h at room temperature. The anterior segment of the eyeball (cornea, iris and ciliary body, and lens) was removed. Retinal tissue was carefully removed from the eyecup while the remaining cup contained RPE, choroid and sclera. Both retinal and RPE/Choroidal flat mounts were thoroughly washed and processed for immunofluorescence staining. 

### 2.8. Immunohistochemistry

Eyes were fixed as described above and processed for 6 μm-thick paraffin sections. Eye sections were dewaxed by xylene and dehydrated by ethanol. Antigen retrieval was then carried out using 0.05% citraconic anhydride buffer in a 95 °C water bath for 30 min. Sections were permeabilized with 0.5% Triton 100X in PBS for 15 min at room temperature and blocked by 5% BSA and 0.1%Triton X in PBS for 1 h. Sections were then incubated with primary antibodies (Table 2) overnight at 4 °C. After thorough washing, samples were incubated with secondary antibody for 1 h at room temperature, washed again thoroughly, mounted by Vectashield mounting medium with DAPI (Vector Laboratories, Newark, CA, USA) and examined by confocal microscopy (Dmi8).

### 2.9. Retinal Cell Quantification, Morphometric Analysis

For immunofluorescence of paraffin sections, 4 images per section from 3 sections per eye were acquired representing the central and mid-peripheral region of the retina. Each section was at least 300 μm away from the previous or following section. Eyes from 4~6 mice were processed for each study group. For whole-mount, retinal areas (measuring 1.272 mm^2^) located 0.5 mm superior, inferior, temporal, and nasal to the optic nerve head were photographed, and whole-mounts from 6 mice were processed for each study group. Fluorescent images were acquired with constant settings for each antibody. Positive cells were quantified using ImageJ 1.48v software (NIH). The data are presented as average ± standard error of the mean (SEM), normalised to 1 mm of retinal length for sections or 1 mm^2^ for whole-mount.

Cone arrestin was used to quantify the number of cone photoreceptor cells. PKCα and secretagogin were representatives of rod-bipolar and cone-polar cells, respectively. NeuN-positive cells in the ganglion cell layer (GCL) represent ganglion cells. IBA-1^+^ or gfp^+^ cells were microglia in *Socs3^fl/fl^* and DKO retina, respectively.

### 2.10. Statistical Analysis

We used the GraphPad Prism analytical program (GraphPad Software, San Diego, CA, USA) for Statistical analysis. Student’s t tests were used for the comparison between two groups. Two-way ANOVA followed by Sidak’s multiple comparison test was used to investigate the effect of minocycline treatment in *Socs3^fl/fl^* or DKO mice. Data are represented as mean ± SEM and significance was established as *p* < 0.05.

## 3. Results

### 3.1. The Effect of Minocycline on Visual Function

**Figure 1:**
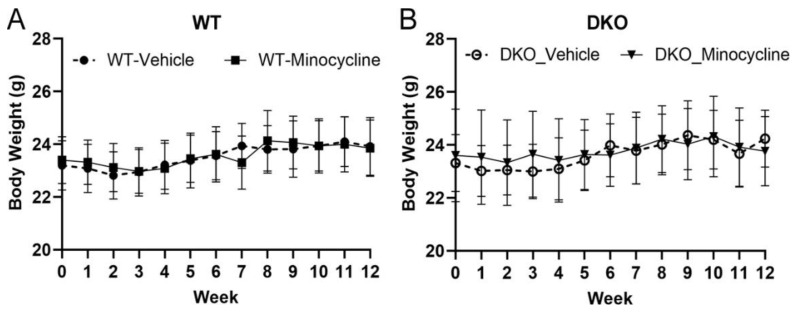
The body weight of wild type (WT, (**A**)) and double knockout (DKO, (**B**)) mice in vehicle or minocycline treated mice during the 3 months study.

The body weight between WT (*Socs3^fl/fl^*) and DKO mice were comparable (Figure 1A,B). Neither WT nor DKO mice showed significant weight loss (Figure 1) or behaviour change during the study. We previously reported ~50% of natural death in DKO mice by 12 months of age [24]. In this study, the vehicle-treated group lost two mice (out of 16) and the minocycline-treated group lost one mouse (out of 12) at 7 months old. None of the WT mice died during the study. We did not observe any difference between males and females in response to minocycline or vehicle treatment.

**Figure 2:**
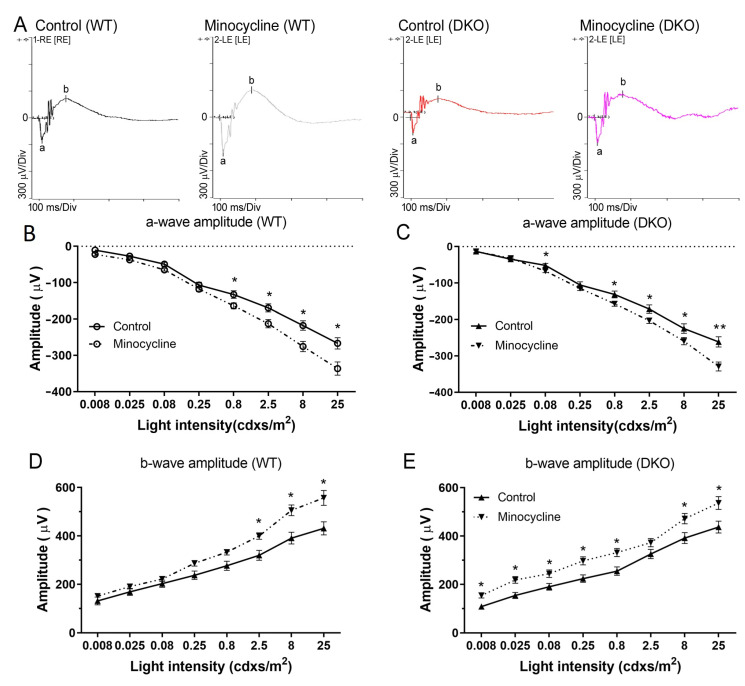
The effect of minocycline on visual function measured by electroretinography (ERG). (**A**) Representative images of scotopic ERG from vehicle-treated or minocycline-treated wild type (WT) and double knockout (DKO) mice. (**B**,**C**) a-wave amplitude of vehicle- and minocycline-treated WT (**B**) and DKO (**D**) mice. (**D**,**E**) b-wave amplitude of vehicle- and minocycline-treated WT (B) and DKO (**D**) mice. Mean ± SEM, *n* ≥ 8 mice (16 eyes), * *p* < 0.05, ** *p* < 0.01 compared to the same light intensity of vehicle-treated mice by 2-way ANOVA with Sidak’s multiple comparison test.

The effect of minocycline treatment on visual function was assessed in both WT and DKO mice using scotopic ERG. The a- and b-wave amplitudes and implicit time were comparable between the vehicle (control) treated WT and DKO mice (Figure 2A). Minocycline treatment significantly improved the a- and b-wave responses in WT and DKO mice (Figure 2). In WT mice, the improvements were only observed in high-light intensity (2.5~25 cdxs/m^2^) (Figure 2B,D). The a-wave improvements in DKO mice were comparable to WT mice. However, minocycline-treated DKO mice had significantly higher levels of b-wave amplitudes in all light intensities compared to vehicle-treated DKO control mice (Figure 2E). We did not observe significant changes in the OP value (data not shown). Our results indicate that minocycline may improve the function of photoreceptors and inner retinal neurons.

### 3.2. The Effect of Minocycline on Retinal Gross Morphology and Thickness

**Figure 3:**
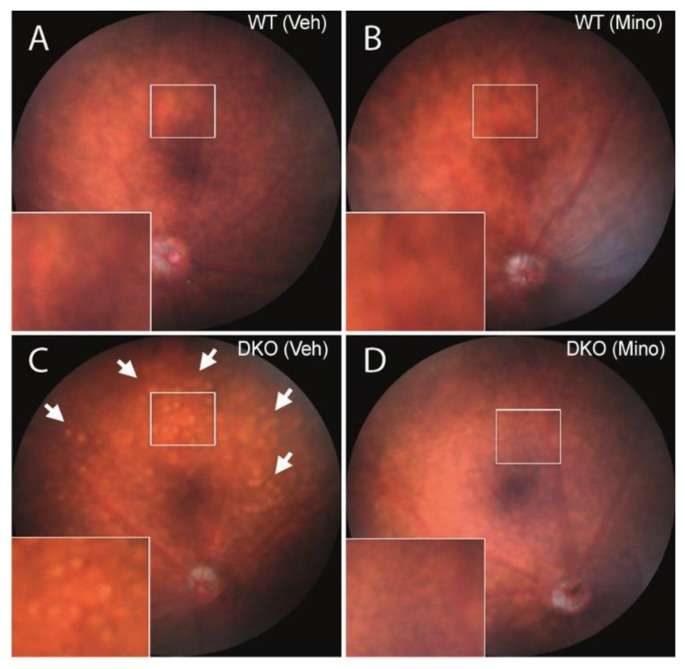
Fundus images of vehicle- or minocycline-treated wild-type (WT) and double knockout (DKO) mice. Fundus images were taken from 9-month-old WT (**A**,**B**) or DKO (**C**,**D**) mice following 3 months vehicle (**A**,**C**) or minocycline (**B**,**D**) treatment. High-magnification images of rectangle areas were shown in the lower left corner of each image. Arrows—whitish dots. Veh—vehicle; Mino—minocycline.

Fundus images showed a normal appearance of blood vessels, optic disc and retinal morphology in vehicle-treated and minocycline-treated WT mice at the age of 9-month-old (Figure 3A,B). Multiple Drusen-like whitish dots were observed in the fundus of vehicle-treated DKO but not minocycline-treated DKO retina (Figure 3C,D). Previous studies reported that these whitish dots were subretinal microglia [25,26]. This suggests that 9-month-old DKO mice exhibited subretinal inflammation, which was suppressed by minocycline treatment.

**Figure 4:**
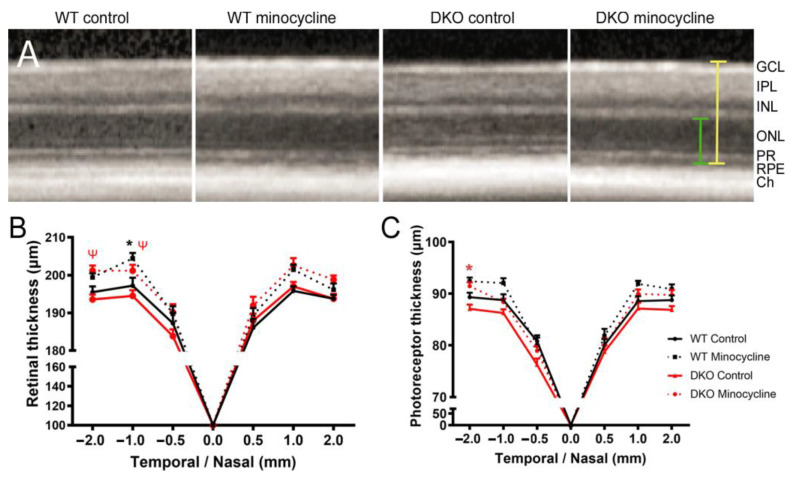
The effect of minocycline on retinal thickness. (**A**) Representative SD-OCT images from vehicle- or minocycline-treated wild type (WT) and double knockout (DKO) mice. Yellow line showing the measurement of the overall retinal neuronal thickness, the green line showing the measurement of outer retina (photoreceptor layer, including ONL and PR) thickness. (**B**,**C**) Graphs showing the overall retinal neuronal thickness (**B**) and photoreceptor layer thickness (**C**) at the positions of 0.5, 1 and 2 mm away from the optic disc at temporal and nasal sites. Mean ± SEM, *n* ≥ 8 mice (16 eyes), * *p* < 0.05 between minocycline-treated and vehicle-treated WT mice at the same location, ^ψ^ *p* < 0.05 between minocycline-treated and vehicle-treated DKO mice at the same location. 2-way ANOVA with Sidak’s multiple comparison test. GCL: ganglion cell layer; IPL: inner plexiform layer; INL: inner nuclear layer; ONL: outer nuclear layer; PR: photoreceptor segments; RPE: retinal pigment epithelia; Ch: choroid; WT: Wild type; DKO: double knockout.

Mouse retinal thickness and the photoreceptor layer including the outer nuclear layer (ONL) and photoreceptor inner/outer segments (PR) thickness were measured in SD-OCT images at 0.5, 1 and 2 mm away from the optic nerve at both nasal and temporal sides. Compared to WT controls, the overall retinal thickness and photoreceptor thickness in DKO mice appeared to be thinner than in WT mice but did not reach statistical significance (Figure 4). Three-month minocycline treatment significantly increased the overall retinal thickness in both WT and DKO mice, particularly in the temporal side 1- and 2-mm distance from the optic disc (Figure 4A,B). The thickness of the outer retina (photoreceptor) was significantly thicker in minocycline-treated DKO mice compared to vehicle-treated DKO mice (Figure 4C). The treatment also appeared to have increased the photoreceptor thickness in WT mice, but the increment did not reach statistical significance (Figure 4C).

### 3.3. The Effect of Minocycline on Retina Microglial Activation

**Figure 5:**
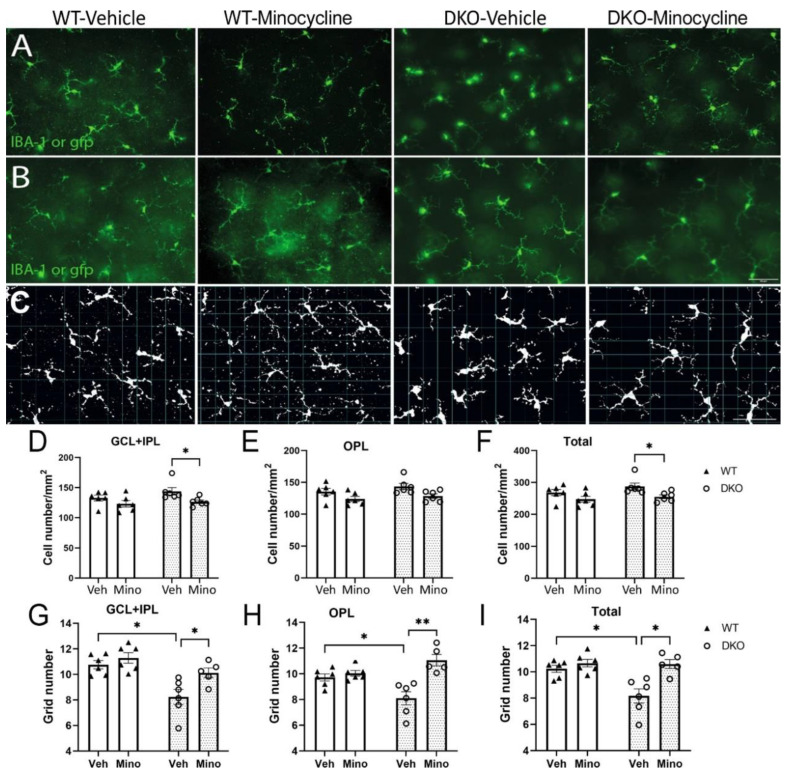
The effect of minocycline in retinal microglial activation. Retinal flatmounts were collected from 9-month-old wild type (WT) or double knockout (DKO) mice following 3 months vehicle or minocycline treatment, and then processed for immunostaining and confocal microscopy. WT retinas were stained for Iba-1. (**A**,**B**) Representative images of microglia in GCL + IPL (**A**) and OPL (**B**) from different groups. Scale bar: 50μm. (**C**) Representative images showing morphological evaluation of microglia from different groups using the grid system. (**D**–**F**) The average number of microglial cells in the GCL+ IPL (**D**), OPL (**E**) and total number of microglia (**F**) in different groups of retinas. (**G**–**I**) The average number of grid coverage of microglia in GCL+ IPL (**G**), OPL (**H**) or both layers (**I**) from different groups. Mean ± SEM, *n* = 6. * *p* <0.05, ** *p* < 0.01, by 2-way ANOVA with Sidak’s multiple comparison test. GCL: ganglion cell layer, IPL: inner plexiform layer; OPL: outer plexiform layer.

Microglia in the WT *Socs^3fl/fl^* mice were identified in retinal flatmounts following Iba-1 staining, whereas microglia in DKO mice were identified based on their gfp expression. The number of microglia in the ganglion cell layer (GCL) + inner plexiform layer (IPL) (Figure 5A) and outer plexiform layer (OPL) (Figure 5B) were counted separately. We found that the number of Iba-1^+^ cells in GCL + IPL and OPL and the total number did not differ between vehicle- and minocycline-treated WT mice (Figure 5A,B,D–F). In DKO mice, the number of microglia in GCL + IPL and the total number were significantly higher in vehicle-treated mice compared with minocycline-treated mice (Figure 5A,B,D–F).

We further analyzed the microglial cell size based on the coverage of dendrites using a grid system previously reported [27] (Figure 5C) as a readout of microglial activation. No significant difference was observed in vehicle- and minocycline-treated WT mice in different retinal layers of microglia (Figure 5G–I). Microglia in both GCL + IPL and OPL from vehicle-treated DKO mice had significantly smaller grid coverage, which was restored in minocycline-treated mice (Figure 5G–I).

**Figure 6:**
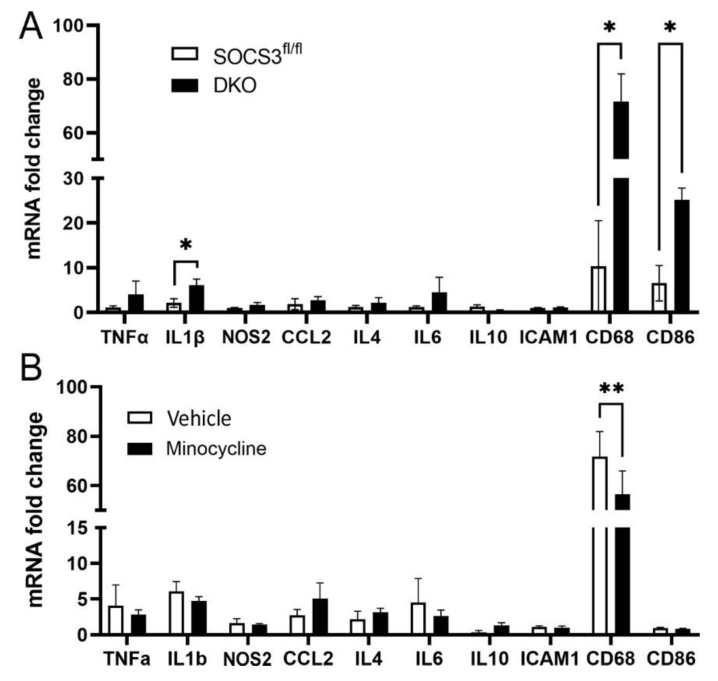
The effect of minocycline in retinal immune related gene expression. The mRNA was extracted from 9-month-old wild type (*Socs3^fl/fl^*) or DKO mouse retinas (**A**) or vehicle- and minocycline-treated DKO (9-month old) retinas (**B**). The expression of inflammatory genes including *Tnfa*, *IL1b*, *Nos2*, *Ccl2*, *Il4*, *Il6*, *IL10*, *Icam1*, *Cd68*, and *Cd86* was evaluated by real-time RT-PCR. Mean ± SEM, *n* = 6. * *p* < 0.05, ** *p* < 0.01 by Student’s *t*-test.

Real-time RT-PCR showed that the DKO retina expressed significantly higher levels of *CD68* and *CD86*. Other genes including *Tnfa*, *IL1b*, *Nos2*, *Ccl2*, *Il4*, *Il6*, *IL10* and *Icam1* did not differ between WT retina and DKO retina (Figure 6A). Three months of minocycline treatment significantly reduced the expression of *CD68* in DKO mice (Figure 6B).

Taken together, our results suggest higher levels of microglia activation in vehicle-treated 9-month-old DKO mice and minocycline treatment reduced the activation.

### 3.4. The Effect of Minocycline on Retinal Neuronal Degeneration in DKO Mice

**Figure 7:**
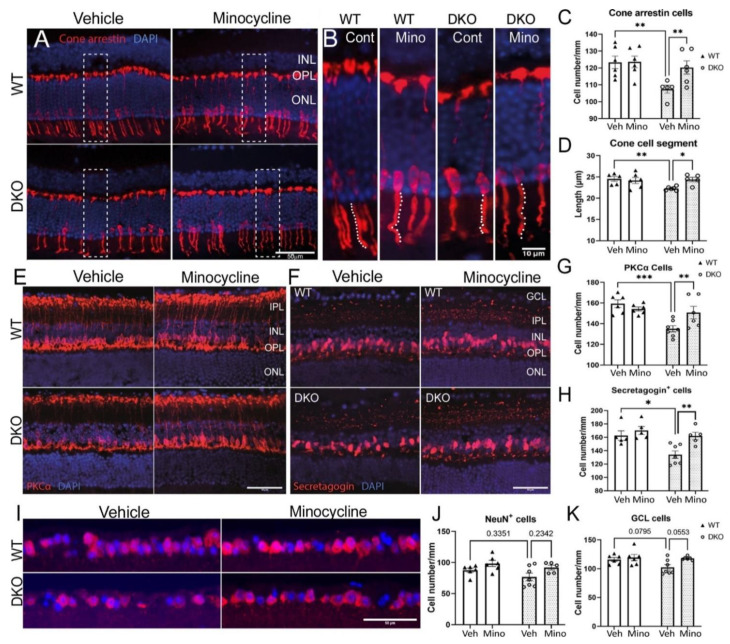
The effect of minocycline on retinal neuronal degeneration. (**A**) Representative images showing cone arrestin^+^ cells from different groups. Cell nuclei were stained with DAPI. High magnification of the rectangle areas is shown in (**B**). White dotted lines in (**B**) illustrate the measurement of inner/outer segment of cone arrestin^+^ cells. (**C**) The number of arrestin^+^ cells in different groups. (**D**) The cone segment length in different groups. (**E**,**F**) Representative images of PKCα^+^ (**E**) and secretagogin^+^ (**F**) cells from different groups. (**G**,**H**) The number of cone arrestin^+^ (**G**) and secretagogin^+^ (**H**) cells in different groups. (**I**) Representative images of NeuN^+^ cells from different groups. Nuclei were stained with DAPI. (**J**) The number of NeuN^+^ cells in different groups. (**K**) The number of DAPI^+^ cells in the ganglion cell layer (GCL) in different groups. Scale bars = 50 µm in (**A**,**E**,**F**,**I**). Mean ± SEM, *n* = 6, * *p* <0.05, ** *p* < 0.01; *** *p* < 0.001 by 2-way ANOVA with Sidak’s multiple comparison test. GCL: ganglion cell layer, IPL: inner plexiform layer; INL: inner nuclear layer; OPL: outer plexiform layer; ONL: outer nuclear layer. Veh: vehicle; Mino: minocycline.

Retinal cone arrestin^+^ cells were quantified by counting the cell number (Figure 7A) as well as measuring the length of cone inner/outer segments (white dotted lines in Figure 7B). There was no difference in the number (Figure 7A,C) and the inner/outer segment length of cone cells (Figure 7B,D) between vehicle- and minocycline-treated WT mice. The number and the segment length of cone cells in 9-month-old control DKO mice were significantly lower than in WT mice, and minocycline treatment significantly increased the number and the segment length of cone arrestin^+^ cells (Figure 7A–D).

The rod bipolar cells were identified by PKCα (Figure 7E) and cone secretagogin was used to identify cone bipolar cells (Figure 7F). The number of PKCα^+^ (Figure 7G) and secretagogin^+^ (Figure 7H) bipolar cells did not differ between vehicle- and minocycline-treated WT mice. Vehicle-treated DKO mice had a significantly lower number of PKCα^+^ (Figure 7G) and secretagogin^+^ (Figure 7H) cells compared to vehicle-treated WT mice and to minocycline-treated DKO mice.

There was some reduction in NeuN^+^ ganglion cells in vehicle-treated DKO mice compared to vehicle-treated WT and minocycline-treated DKO mice although the difference did not reach statistical significance (Figure 7I,J). A similar trend was observed in the total number of DAPI^+^ cells in the RGL (Figure 7I,K). Nonetheless, our results suggest that minocycline did not affect the number of ganglion cells in WT and DKO mice.

Our data suggest that the DKO mice had significant bipolar cell and photoreceptor degeneration by 9 months old, and minocycline treatment (starting from 5 months of age) effectively prevented retinal degeneration.

### 3.5. The Protective Effect of Minocycline on RPE Damage in DKO Mice

**Figure 8:**
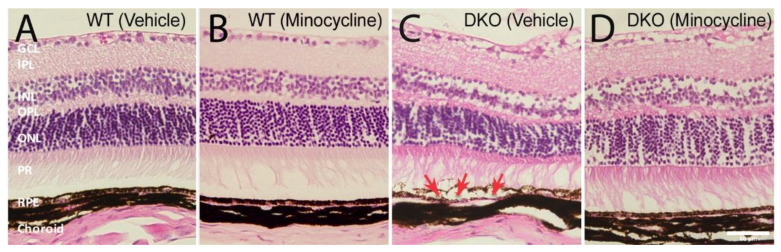
The effect of minocycline on retinal structure in histological examination. Representative images of hematoxylin and eosin (H&E) stained retinal sections from vehicle-treated wild type (WT) (**A**), minocycline-treated WT (**B**), vehicle-treated double knockout (DKO) (**C**) and minocycline-treated DKO mice (**D**). Arrows in (**C**) indicating RPE swelling and vacuoles. GCL: ganglion cell layer; IPL: inner plexiform layer; INL: inner nuclear layer; OPL: outer plexiform layer; ONL: outer nuclear layer; PR: photoreceptor segments; RPE: retinal pigment epithelium. Scale bar = 50 μm.

We previously showed that the aged DKO mice (10~12 months old) developed sub-retinal microglial accumulation and localised RPE damage [28]. In this study, histological investigations showed normal retinal structure in the vehicle- and minocycline- treated WT mice (Figure 8A,B). The vehicle treated DKO mice displayed discrete areas of RPE vacuoles and corresponding distorted retinal layers (arrows in Figure 8C). The RPE damage was not observed in minocycline treated DKO mice (Figure 8D).

**Figure 9:**
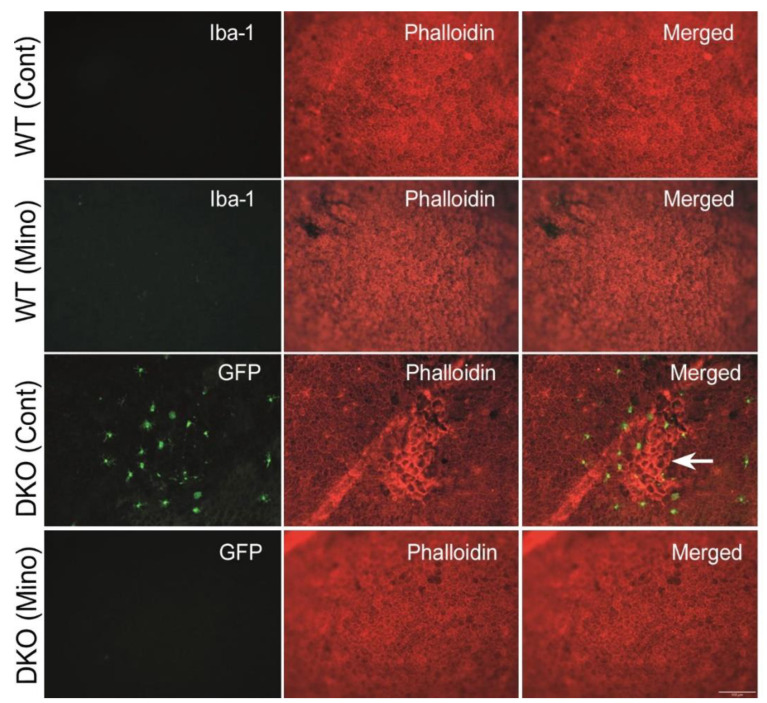
The effect of minocycline on the structure of retinal pigment epithelial cells. RPE/choroidal flatmounts were stained with phalloidin (red) and Iba-1 (green, for wild-type mice). Microglia in double knockout (DKO) mice were GFP^+^. Arrow indicating RPE dysmorphia and microglial accumulation. Scale bar = 100 μm. RPE: retinal pigment epithelium.

We further investigated RPE structure in flatmount examination following phalloidin (and Iba-1 in WT mice) staining. No RPE dysmorphia was observed in the vehicle and minocycline treated WT mice (Figure 9), and Iba-1^+^ cells were rarely detected. Vehicle-treated DKO mice displayed patches of RPE dysmorphia accompanied by infiltrating GFP^+^ microglial cells, which was not observed in minocycline-treated DKO mice (Figure 9).

## 4. Discussion

In this study, we show that minocycline effectively suppressed microglial activation and preserved visual function in a mouse model of chronic retinal degeneration, the *LysMCre-Socs3^fl/fl^Cx3cr1^gfp/gfp^* DKO mice. In this model, two immune checkpoint genes *Cx3cr1* and *Socs3* were deleted in microglia leading to an unchecked age-related immune response to oxidative insults and dysregulated retinal para-inflammation. This low-grade chronic inflammation resulted in multiple types of retinal neuronal degeneration and impaired visual function by 10–12 months of age [24]. The model mirrors the clinical pathological time course, whereby retinal degeneration is caused by sustained low-grade chronic inflammation, and the disease progresses slowly over many years, such as age-related macular degeneration, diabetic retinopathy, and retinitis pigmentosa.

The therapeutic effect of minocycline in retinal degeneration (e.g., various RD mice [20,23] and light-induced models [29,30]) has been studied extensively in recent years. However, only short-term (1–2 weeks) effects were reported in these studies, whereas clinical management of these diseases requires long-term (even life-long) management. Therefore, it is necessary to test the therapeutic potential of minocycline in a chronic model of retinal degeneration. In a previous study, we found that a proportion of the DKO mice showed signs of slower and limited movement at 7~8 months old and some of them died before 12 months old [24]. This indicates the existence of detrimental inflammation a few months before 7–8 months of age. Therefore, in this study, we started the minocycline treatment in 5-month-old DKO mice and continued the treatment for 3 months. One human year is equivalent to 2.6 adult mouse days [31]. Three months of treatment in mice is equivalent to ~35 years in human beings. Our results suggest that long-term minocycline treatment is safe and effective in treating retinal degeneration.

Inflammation is a protective response to tissue injury and essential to remove dead cells/debris and promote tissue repair, although it can cause further damage when the response is dysregulated or becomes too severe. In a general health context, sustained immune suppression may dampen the beneficial role of inflammation and worsen retinal degeneration, for example, by allowing dead cells to accumulate at the lesion site. On the other hand, long-term anti-inflammatory treatment may also increase the risk of infection and cancer development, as seen in patients with autoimmune diseases who require prolonged steroid treatment. Minocycline was originally discovered as an antibiotic due to its ability to bind to the bacterial 30S ribosomal subunit and inhibit protein synthesis [32]. Recent studies have shown that it can suppress inflammation by inhibiting NFkB activation [33]. It has been shown that minocycline can suppress LPS-induced microglial activation in vitro as well as in various animal models of brain injury [34] and retinal degeneration [35], including disease-associated microglia (DAM) [36]. In the current study, microglia were activated by naturally occurring age-related oxidative insults and we observed that age-related microglial activation was exacerbated to vision worsening levels, due to the lack of CX3CR1 and SOCS3 in the DKO mice. Furthermore, we found that minocycline can prevent this exacerbation of vision loss and retinal degeneration. CX3CR1 deliver a “do not activate” signal to microglia once engaged with neuron-derived CX3CL1 [37,38]; whereas SOCS3 negatively regulate the JAK2/3-STAT3 pathway of the cytokine receptor signalling cascades [39,40]. Our results suggest that minocycline can substitute the absence of checkpoint proteins of the cytokine receptor activation signalling pathways in DKO microglia cells and prevent deleterious effects to the retina.

Minocycline is also reported to be neuroprotective (independent of its anti-inflammation role) [41]. This may be related to its anti-apoptotic and anti-oxidative functions through activating the Nrf2 pathway [40] and the ability to act as a matrix metalloproteinase (MMP) inhibitor [42]. The neuroprotective effect was observed in both the WT and DKO mice in this study evidenced by improved visual function (i.e., higher a- and b-wave amplitudes of ERG response). Since WT mice presented no evidence of retinal neuronal loss and microglial activation at 8 months of age, our results suggest that minocycline can improve neuronal function.

A previous study reported that minocycline at low concentrations (50 nM~20 µM) stimulated chemotaxis and decreased RPE cell proliferation, and at high concentrations (>5 µM) induced RPE cell necrosis [43]. In our study, 3 months of minocycline (25 mg/kg, daily) treatment at the dose approved for clinical use did not cause any RPE dysmorphology in WT and DKO mice. Instead, microglia mediated RPE damage in DKO mice was prevented by long-term minocycline treatment. Our results highlight the importance of in vivo verification of the in vitro results.

The study’s strengths include the use of a novel model of age-related retinal degeneration with proper controls and sufficient animal numbers, the combination of clinical (ERG and OCT) and post-mortem laboratory investigations, and a thorough investigation of retinal neurons and different layers of microglial cells. However, the study is observational and does not provide mechanistic insights. Future studies using microglia and neurons from the DKO mice will help to understand the mechanism of minocycline-mediated immune suppression and neuroprotection.

## 5. Conclusions

In summary, we show in this study that three months of minocycline treatment improved visual function in both WT and DKO mice and effectively suppressed microglial activation, reduced neuronal death, and protected RPE cells in DKO mice. Our results suggest that minocycline, the clinically approved drug, can be re-purposed as a long-term therapy to control chronic microglial activation and treat retinal degeneration although further studies are needed to evaluate its safety and efficacy in patients.

## Figures and Tables

**Table 1:** Primer sequences for qRT-PCR.

Genes	Forward	Reverse
*Actb*	GGCACCACACCTTCTACAATG	GGGGTGTTGAAGGTCTCAAAC
*Tnfa*	TCTCATGCACCACCATCAAGGACT	ACCACTCTCCCTTTGCAGAACTCA
*Nos2*	TCTTTGACGCTCGGAACTGTAGCA	ACCTGATGTTGCCATTGTTGGTGG
*Icam1*	CACGTGCTGTATGGTCCTCG	TAGGAGATGGGTTCCCCCAG
*Il1b*	AAGGGCTGCTTCCAAACCTTTGAC	ATACTGCCTGCCTGAAGCTCTTGT
*Il4*	ACGGAGATGGATGTGCCAAAC	AGCACCTTGGAAGCCCTACAGA
*Il6*	ATCCAGTTGCCTTCTTGGGACTGA	TAAGCCTCCGACTTGTGAAGTGGT
*Il10*	GGCAGAGAACCATGGCCCAGAA	AATCGATGACAGCGCCTCAGCC
*Cd68*	TTGCTAGGACCGCTTATAG	AAGGATGGCAGGAGAGTA
*Cd86*	TCTCCACGGAAACAGCATCT	CTTACGGAAGCACCCATGAT
*Ccl2*	GCATCCACGTGTTGGCTCA	CTCCAGCCTACTCATTGGGATCA

**Table 2:** Primary and secondary antibodies used in immunofluorescence study.

Antigen	Dilution	Company	Host
IBA-1	1:200	Wako Chemicals, Osaka, Japan	Rabbit
Cone arrestin	1:1000	Millipore, Temecula, CA, USA	Rabbit
PKCα	1:500	Santa Cruz, Santa Cruz, CA	Rabbit
Secretagogin	1:500	Biovendor R&D, Brno, Czech Republic	Sheep
NeuN	1:100	ThermoFisher, Waltham, MA, USA	Rabbit
**Marker**			
Alexa Fluor™ 594 Phalloidin	1:100	ThermoFisher, USA	F-actin probe
**Secondary Antibody**			
Alexa Fluor 594	1:400	Invitrogen, San Diego, CA, USA	Donkey anti-rabbit
Alexa Fluor 594	1:400	Invitrogen, USA	Donkey anti-sheep
Alexa Fluor 488	1:400	Invitrogen, USA	Donkey anti-rabbit

## Data Availability

The data presented in this study are all contained within the main body of this article.
